# Is mother-to-infant transmission the most important factor for persistent HBV infection?

**DOI:** 10.1038/emi.2015.30

**Published:** 2015-05-20

**Authors:** Zixiong Li, Xiaomei Hou, Guangwen Cao

**Affiliations:** Department of Epidemiology, Second Military Medical University, Shanghai 200433, China

**Keywords:** hepatitis B virus, mother-to-child transmission, chronic infection

## Abstract

Of the infants born to hepatitis B surface antigen (HBsAg)-positive mothers globally, 42.1% who did not receive hepatitis B virus (HBV) passive-active immunoprophylaxis and 2.9% of infants who received the immunoprophylaxis acquired HBV infection perinatally. Moreover, perinatal infection occurred in 84.2% (18.8%–100%) and 8.7% (0.0–21.0%) of infants born to hepatitis B e-antigen (HBeAg)-positive mothers who did not and did receive immunoprophylaxis, respectively; by contrast, the infection rates were 6.7% (0.0–15.4%) and 0.4% (0.0–2.5%) for infants born to HBeAg-negative-carrier mothers, respectively. The chronicity rates of HBV infection acquired perinatally were 28.2% (17.4%–33.9%) in infants born to HBeAg-negative mothers and 64.5% (53.5%–100%) in infants born to HBeAg-positive mothers. HBV mother-to-child transmission was more frequent in East Asia relative to other areas. In addition to differences in the endemic HBV genotype, the interchange of allelic dominance in genetic polymorphisms in HLA class II and NF-κB between the Chinese and European populations may explain why chronic HBV infection frequently affects the Chinese. The risk of progressing into chronic infection was inversely related to the age of children at the time of horizontal transmission. To further diminish HBV chronic infection, it is necessary to enforce antiviral treatment after the 28th week of gestation for HBeAg-positive mothers and to improve the health habits of carrier mothers and household sanitary conditions.

## INTRODUCTION

Chronic hepatitis B virus (HBV) infection, defined as a positive test for the hepatitis B surface antigen (HBsAg) for six months or more, is one of the leading public health problems worldwide and contributes to the development of hepatocellular carcinoma (HCC). Mainland China alone accounts for one-fifth of the global population, one-third of the global HBV carriers, and half of the global HCC cases.^1,2^ More than 90% of HCC cases diagnosed in mainland China are positive for HBsAg and/or circulating HBV DNA (Yang F *et al.*, unpublished data (2015)). Based on the endemicity of HBV genotypes in mainland China, the HCC-promoting effect of HBV genotype C (vs. genotype B) and the incidence of HCC in the population seropositive for HBsAg in Taiwan,^1,3,4^ we estimate that approximately 32% of male and 9% of female chronic HBV-infected subjects in mainland China will develop HCC before they reach 75 years of age. HCC is a highly fatal disease, with a five-year survival rate of 9% for patients who do not receive surgical treatment and 33% for those who receive curative surgery according to our recent community-based epidemiological study in Shanghai, mainland China.^5^ A reduction in the prevalence of chronic HBV infection is indispensable for reducing the HCC burden. Understanding the mechanism by which HBV causes chronic infection may pave the way for effective prophylaxis and treatment of the liver diseases caused by the infection.

HBV infection acquired in early childhood is typically considered to be one of the major causes of chronic infection. It has been frequently reported by respected academics and clinicians that the likelihood of developing chronic HBV infection is approximately 90% in individuals who are infected perinatally; additionally, over 90% of infected infants are reported to follow a chronic course after HBV is acquired via mother-to-child transmission (MTCT) in endemic areas.^6,7,8,9,10^ MTCT of HBV has been postulated to account for more than half of chronic infections in highly endemic areas.^9^ Early evidence supporting the statement of this “90%” possibly came from a randomized blind controlled clinical trial performed in Taiwan. In this clinical trial, infants of hepatitis B e-antigen (HBeAg)-positive HBsAg-carrier mothers were given hepatitis B immune globulin (HBIG) immediately after birth, followed by one of three HBV vaccination schedules. Persistent HB antigenemia occurred in only nine (6%) of the 159 infants receiving prophylaxis but in 88% of the controls.^11^ However, the percentage of seropositivity for HBeAg in HBsAg-carrier mothers depends on other factors, including the age of gestation, the circulating level of HBV DNA, and the HBV genotype/subgenotype. In our recent study performed in Shanghai and Zhejiang province, mainland China, only 30% of HBsAg-carrier women were seropositive for HBeAg at gestation.^12^ Thus, it is difficult to believe that approximately 90% of infants infected perinatally with HBV follow a chronic course of infection. In this article, three questions will be addressed based on updates from formal publications: first, what are the percentages of infants who acquire HBV infection perinatally from their carrier mothers, and what is percentage of those with HBV MTCT progress into chronic infection in the pre- or postimmunization era? Second, what is the major reason that persistent HBV infection frequently affects the Chinese population? Third, what are practicable steps to minimize the possibility of chronic HBV infection?

## TRANSPLACENTAL TRANSMISSION OF HBV AND INFLUENCING FACTORS

Familial aggregation of HBV infection was demonstrated as early as 1966, when HBsAg was first reported by Dr Blumberg.^13,14^ Although father-to-child transmission was evident by phylogenetic analysis of the HBV genome, MTCT of HBV was believed to be a major intrafamilial transmission pattern and the development of HCC in the offspring was more related to HBV infection in the mother than in the father.^15,16^ MTCT of HBV can be vertical (transplacental transmission, also termed as intrauterine HBV infection) or can occur horizontally early in the postpartum period. In some HBV-endemic areas (i.e., West Africa), transplacental transmission of HBV seems to be very rare.^17,18^ However, in East Asia (i.e., Taiwan) where genotype B is endemic, transplacental transmission of HBV is frequently evident, especially in infants whose mothers are seropositive for HBeAg.^11^ In mainland China, 27.2% of families were found to have one or more HBsAg-positive members, and a strong tendency for family clustering was identified.^19^ Horizontal transmission was reported to be an important route of HBV infection during early childhood; contact with infected family members probably accounts for the majority of horizontal transmission in children. The proportion of chronic HBsAg carriage attributable to perinatal transmission has been estimated to be only 13%–20%.^19^ The presence of HBV DNA in fetal cord blood indicates the exposure of the fetus to HBV, while HBsAg positivity in infants of six months of age may indicate the establishment of HBV infection. Intrauterine HBV infection should be defined if the neonatal blood test is positive for HBsAg and/or HBV DNA. However, compliance with the collection of fetal peripheral blood at birth is actually very poor. HBV serological markers (i.e., HBV DNA) in the maternal peripheral blood and fetal cord blood are suitable to determine HBV transplacental transmission. However, because fetal cord blood is likely to be contaminated by maternal blood during delivery process, cord blood should be sampled with a syringe after aseptic washing with normal saline to avoid possible contamination with maternal peripheral blood. Using this method, we collected 537 maternal peripheral blood-cord blood pairs to investigate HBV transplacental transmission. We found that 143 (26.6%) of the 537 pairs were positive for HBsAg and 52 (9.68%) were positive for HBV DNA (within the detection limit of 500 copies/mL).^12^ HBV DNA appeared more frequently in fetal cord blood from mothers seropositive for HBeAg than in blood from HBeAg-negative mothers (26.09% vs. 2.66%, *P* < 0.001). A similar study performed in Nigeria also indicated that the rate of transplacental transmission of HBsAg was 24%, and high maternal HBV DNA levels were associated with increased neonatal HBV-DNA titers (*P* = 0.001).^20^ High maternal HBV DNA levels contribute greatly to HBV transplacental transmission. Compelling epidemiological evidence supports the hypothesis that factors including maternal blood seropositive for HBeAg, high maternal circulating HBV DNA (>10^6^ copies/mL), a high maternal HBsAg titer, HBV genotype B2 (vs. C2), male fetus, amniocentesis, pregnancy complications such as threatened preterm labor or prolonged labor, maternal menstrual irregularity, severe nausea during the first trimester, the presence of HBsAg or HBV DNA in fetal umbilical cord blood, and antigenemia in siblings greatly increase the risk of transplacental transmission of HBV.^12,21,22,23,24,25,26,27,28^ Maternal HBeAg positivity and high circulating HBV DNA may be the most important of these risk factors. The presence of HBeAg in the maternal sera correlates well with high titers of HBsAg and HBV DNA.^12,26^ A survey performed in a cohort of Korean-Americans without HBV vaccination indicated that the vertical transmission rate was 30.3% in children born to HBsAg-positive mothers and 100% in those born to HBeAg-positive mothers.^29^ Transplacental maternal HBeAg may induce immunologic tolerance *in utero*, thereby facilitating intrauterine HBV infection.^30^ Thus, the expression of HBeAg may represent a viral strategy to guarantee persistence after perinatal infection because it induces specific unresponsiveness of helper T cells to HBcAg and HBeAg in the neonates born to HBeAg-positive-carrier mothers.^31^ Antiviral treatment using lamivudine and possibly other nucleot(s)ide analogs from the 28th week of gestation is the most effective and safe method to reduce transplacental transmission from mothers with high levels of viremia; elective cesarean section might also be effective in reducing transplacental transmission of HBV.^21,22,23,32^ These data should be useful for the prophylaxis of HBV transplacental transmission. Pregnant women with circulating HBV DNA titers ≥10^6^ copies/mL should be considered for active prophylactic strategies, such as antiviral treatment for carrier mothers during the late stage of pregnancy, to reduce the risk of transplacental transmission.

## PERINATAL HBV TRANSMISSION BEFORE THE WIDESPREAD APPLICATION OF THE HBV VACCINATION PROGRAM

Transplacental transmission of HBV indicates the exposure of the infants to HBV but does not necessarily reflect active HBV infection in the infants' hepatocytes. Because HBeAg and IgG antibodies (i.e., anti-HBc and anti-HBs) are transplacental, even the detection of IgG and HBeAg antibodies in the infants' sera within six months after birth cannot be used to indicate HBV infection. It is also unsuitable to indicate HBV infection in infants using circulating HBV DNA because the cutoff for the qualitative analysis of HBV DNA depends on the method used. This cutoff was unstable in early studies. It is reasonable to indicate the presence of infant infection using serum HBsAg because this parameter can reflect the synthesis of HBV in the infants' hepatocytes. We propose that early HBV infection is established if an infant is seropositive for HBsAg at the age of 0–6 months.

Data from early studies prior to the widespread application of the HBV vaccination program (e.g., prior to 1984 in Taiwan, 1987 in Shanghai, and 1992 in the rest of China) or in low-endemic areas without an HBV vaccination program are important to address the natural process of the chronic progression of HBV infection acquired in early childhood. In an early study performed in Taiwan, HBV antigenemia developed in 63 of 158 babies (39.9%) born to HBsAg-carrier mothers; a total of 51 (81.0%) of the 63 antigenemic babies became HBsAg-positive within the first six months of life.^24^ A total of 88% of the control infants became HBsAg-positive in the former clinical trial testing for HBV vaccination prophylaxis.^11^ An early study performed in Singapore indicated that 19 of 27 (70.4%) infants perinatally infected with HBV from carrier mothers remained HBsAg-positive, while seven (25.9%) lost the antigenemia and acquired anti-HBsAg antibodies at one year of age.^26^ A study performed in the Philippines found that 7 of 17 (41.2%) infants born to HBsAg-carrier mothers became HBsAg-positive within the first 12 months of life. The risk was higher if the mother was positive for both HBsAg and HBeAg (odds ratio (OR) = 91.0) compared to infants whose mothers were positive for both HBsAg and anti-HBe antibody.^33^ The rate of chronic progression was reported to be as high as 78.4% at an age of 14 months in vaccination-free infants born to carrier mothers positive for HBsAg and HBeAg.^34^ Thus, maternal HBeAg contributes greatly to the chronic progression of HBV infection in their infants.

## EFFECT OF HBV VACCINATION ON PERINATAL HBV TRANSMISSION

Importantly, the occurrence of HBV MTCT differed significantly before and after the HBV vaccination program applied under the recommendation of the World Health Organization (WHO). According to the WHO program, three-dose recombinant vaccines (5 µg/dose, now 10 µg/dose) are administered at 0, 1, and 6 months after birth, while HBIG is administered within 24 h after birth to newborns born to HBeAg-positive, HBsAg-carrier mothers. Standard HBV vaccination with or without HBIG for newborns born to HBeAg-positive mothers should affect the rate of chronic HBV progression. HBV vaccination combined with HBIG for newborns born to HBeAg-positive mothers can reduce the chronic progression of HBV infection acquired perinatally. A study including 109 HBsAg-positive mothers and their newborns in Greece indicated that although HBV-DNA levels were significantly higher in newborns of HBeAg-positive mothers, no child developed chronic HBV infection following the administration of active–passive immunoprophylaxis.^35^ A study performed in Gambia indicated that vaccination was 84% effective against infection and 94% effective against chronic carriage in three- to four-year-old children who had received the vaccine in infancy. Although highly effective in preventing HBV MTCT, standard passive–active immunoprophylaxis with the HBV vaccine and HBIG may have a failure rate as high as 10%–15%.^36^ Vaccinated infants of mothers positive for both HBsAg and HBeAg were at greater risk of breakthrough infection and chronic carriage than infants from uninfected mothers.^37^ In Taiwan, where a standard HBV vaccination program has been performed since 1984, the overall breakthrough infection rate (defined by anti-HBc positivity at more than 24 months of age and including HBsAg positivity) was 5.52% in all of the children born to the HBsAg-carrier mothers; the rate was much higher in children born to the HBeAg-positive mothers than in children born to HBeAg-negative mothers (16.76% vs. 1.58%, *P* < 0.001). The rate of chronicity was also proven to be higher among children born to HBeAg-positive mothers relative to children with HBeAg-negative mothers (54% vs. 17%; *P* = 0.002). Administration of HBIG to infants born to HBeAg-negative mothers did not appear to reduce the rate of chronic HBV infection.^38^ In our previous study, we found that all infants with breakthrough infection were born to HBeAg-positive mothers with circulating HBV DNA levels >10^6^ copies/mL and detectable cord blood HBV DNA.^12^ A study performed in Chinese mothers indicated that maternal HBV DNA levels and detectable HBV DNA in the cord blood were independent risk factors for the failure of passive–active immunoprophylaxis.^39^ A prospective study performed in West China with 214 infants born to HBsAg-positive mothers who received standard passive–active immunoprophylaxis demonstrated that MTCT occurred in 4.7% (10/214) of the infants. Of those, only two (0.9%) infants evolved to carrier status after 36 months of follow-up.^40^ A similar study performed in South China where the subgenotypes B2 and C1 are endemic showed that 7.4% (7/95) of children were infected with HBV during their first year of life despite receiving passive–active immunoprophylaxis. HBeAg was present at higher titers in the birth sera of the babies with HBV breakthrough infection compared to the babies without breakthrough infection; moreover, the mean serum HBV DNA levels in the mothers of the four infants with HBV breakthrough infection was significantly higher than in the mothers of babies who did not become infected.^41^ In South Korea where subgenotype C2 is endemic, 144 children born to HBsAg-seropositive mothers received the passive–active immunoprophylaxis. Seventeen (11.8%) of these infants suffered immunoprophylaxis failure; this failure was significantly associated with maternal HBeAg-seropositivity and HBV DNA seropositivity.^42^ In Iran where HBV genotype D is endemic, a study that enrolled 235 infants born to HBV-infected mothers who received passive–active immunoprophylaxis indicated that HBsAg was detected in six (17.6%) infants between 12 and 15 months of age born to HBeAg-seropositive mothers and three (1.5%) infants born to anti-HBe-seropositive mothers (*P* = 0.0001).^43^ Perinatal transmission and the maternal viral load that facilitate HBV MTCT are important risk factors for hepatocarcinogenesis in children.^44,45,46^ Importantly, children with breakthrough infection have a higher risk of developing HCC compared with nonvaccinated HBV-carrier children.^47^ Although the chronic progression of HBV infection acquired perinatally differs greatly among populations or races infected with different HBV genotypes or subgenotypes, maternal HBeAg and circulating HBV DNA are the most important risk factors for the chronic progression of HBV infection.

## THE PERCENTAGES OF PERINATAL HBV TRANSMISSION BEFORE AND AFTER IMMUNOPROPHYLAXIS

Table 1 summarizes the percentages of HBV MTCT before and after immunoprophylaxis in different populations worldwide based on published studies.^11,12,17,18,20,24,29,33,35,38,39,40,41,42,43,48,49,50,51,52,53,54,55,56,57,58,59^ Globally, the HBsAg-positive rate was 16.2% (ranging from 0.0 to 72.0%) in the cord blood of infants born to carrier mothers. The positive rate was 42.1% (from 0.0 to 100%) in infants born to carrier mothers without active–passive immunoprophylaxis and 2.9% (from 0.0 to 21.0%) in infants born to carrier mothers with active–passive immunoprophylaxis. These data indicate that postpartum infection contributes to HBV infection in infants, while active–passive immunoprophylaxis effectively reduces HBV infection in infants. In East Asia, transplacental HBV transmission occurred in 10.4% (0.0–21.0%) of infants born to HBeAg-negative-carrier mothers; this percentage rose to 21.8% (14.3%–39.8%) in infants born to HBeAg-positive-carrier mothers. Following active–passive immunoprophylaxis, approximately 0.4% (ranging from 0.0 to 2.5%) of infants born to HBeAg-negative-carrier mothers acquired HBV infection perinatally; this percentage rose to 8.5% (4.9%–21.0%) if the mother was HBeAg-positive. Thus, in addition to the current active–passive immunoprophylaxis, we suggest that antiviral treatment should be given to carrier mothers seropositive for HBeAg during the late stage of pregnancy to block the intrauterine transmission of HBV.

## PROGRESSION OF PERINATAL HBV INFECTION INTO CHRONIC INFECTION

The definition of perinatally acquired infant HBV infection has not been consistent in the published literature. We included published studies in which infant HBV infection was defined as the presence of a neonatal blood test positive for HBsAg and/or HBV DNA or HBsAg positivity at six months old of age because infant HBsAg positivity at birth has the highest sensitivity in predicting intrauterine HBV infection, while neonatal HBsAg and HBV DNA double positivity have the highest specificity.^56^ Based on published data and our own study, we calculated the rate of perinatally acquired HBV infection that progressed into chronic infection. Table 2 summarizes the chronic progression of HBV infection in infants established before six months of age based on published studies.^24,26,33,38,40,56,57,58,60,61^ The chronic transformation of HBV infection acquired from birth to six months of age was 28.2% (17.4%–33.9%) in infants whose mothers were seronegative for HBeAg and 64.5% (53.5%–100%) in infants whose mothers were seropositive for HBeAg.

## IMPORTANCE OF POSTNATAL HBV INFECTION TO CHRONIC PROGRESSION

Based on the phylogenetic analysis of whole HBV genomic sequences, we determined that at least 22.1% of 1- to 15-year-old children acquired HBV infection from sources other than their mothers,^12^ indicating that close person-to-person contact with HBV carriers probably represents an important method of HBV transmission in early childhood even in the postimmunization era. Although perinatal HBV transmission was not evident in a study performed in Senegal, six of 34 babies born to HBsAg-positive and HBeAg-negative mothers became infected with HBV, and four of the six developed chronic infections in the second year of life. Infections occurred at a higher rate in infants born to HBsAg-positive (17%) than to HBsAg-negative (4%) women during the first three years of life.^17^ One interesting study showed that 16 of 17 infants born to HBsAg-positive mothers were negative for HBsAg at birth; a total of 66.7% of the infants became HBsAg-positive after an average time of 48 days. Elimination of HBsAg occurred in seven infants, indicating that neonatally infected children are capable of actively eliminating HBV.^62^ An early study performed in Taiwan showed that 10.6% of 924 HBV-free children (mean age: 29 months) who were followed for an average of 2.1 years seroconverted for HBV markers, with an annual incidence of 5.0%. Among the 98 children who experienced HBV infections during the study, 23% became HBsAg carriers. The HBsAg persistence was age-related, with most carriers among the youngest children infected.^63^ The incidence of HBV infections during the second and third year of life was determined for 105 children born to HBsAg-carrier mothers in Taiwan. Infants negative for HBV markers at 12 months of age were followed for an average of 17.5 months, and 38.1% of the infants became infected. The rate was highest for children whose mothers were positive for HBeAg (57.1%), moderate for those whose mothers were negative for both HBeAg and anti-HBe (20.4%), and lowest for those whose mothers were positive for anti-HBe (11.3%).^60^ Close contact with carrier mothers with high viral loads may be the major reason for HBV infections in early childhood. Maternal health habits and/or household sanitary conditions might represent a major contributing factor to the horizontal transmission of HBV from mother to child. A study performed in Yupik Eskimos of southwestern Alaska demonstrated that 28.8% of patients who were four years of age or younger when infected with HBV became chronic HBV carriers compared with 7.7% of patients who were 30 years of age or older when they acquired the infection.^64^ In adults, acute clinical hepatitis B seems to be a self-limiting disease that rarely leads to the development of the carrier state.^65,66^ In our previous study in Shanghai, mainland China, 8.5% of adult patients with acute hepatitis B developed a chronic carrier state; subgenotype C2 HBV was proven to be a unique independent risk factor (OR = 6.97) for chronic progression.^67^ Table 3 summarizes the chronic progression of HBV infection acquired from birth to adulthood based on published studies.^17,64,68^ These data indicate that the risk of becoming a chronic HBV carrier is inversely related to the age of the subject at the time of infection. Such infections can be prevented by the use of HBV vaccination alone; the administration of HBIG should not be necessary. We summarized data from several large population-based studies that indicated that the ratios of vertical transmission and horizontal transmission of HBV acquired in chronic infection changed greatly due to the HBV vaccination program. The vaccination program greatly reduced the ratio of horizontal transmission in the composition of subjects with chronic HBV infection (Table 4) based on published studies.^19,47,69,70,71^

## EFFECT OF HBV GENOTYPES AND MUTATIONS ON THE CHRONIC PROGRESSION OF HBV INFECTION ACQUIRED IN EARLY CHILDHOOD

A total of 10 HBV genotypes (A–J) have been identified based on a sequence divergence of >8% across the entire HBV genome and >4% in the preS/S region of the HBV genome. The HBV vaccine was developed using the surface antigen encoded by the S region of the HBV genome. An antibody to the HBs antigen (anti-HBs) can neutralize HBV infectivity. The commercially available recombinant HBV vaccines are mostly developed based on HBV genotype A (patented by Merck). However, there is >4% nucleotide divergence in the S region among HBV genotypes,^72^ which may contribute to the relatively weak prophylactic effect of the vaccine for HBV genotypes B and C that are endemic in most of Asia. An interesting study performed in Taiwan indicated that HBsAg-carrier children born to HBsAg-positive mothers following standard HBV vaccination had a higher likelihood of genotype C HBV infection compared to unimmunized children (OR = 3.03, *P* = 0.001). By contrast, the increased genotype C to genotype B ratio was not observed in the HBsAg-carrier mother pool in the postimmunization era, indicating that immunized children born to genotype C HBV-infected mothers have a higher rate of breakthrough infection than those born to genotype B HBV-infected mothers.^73^ In our previous population-based epidemiological studies, we found that genotype B was more apt to be cleared by antiviral immunity than genotype C.^1,67^ An animal study revealed three phenotypes of HBeAg/HBcAg-specific T-cell tolerance: (i) profound T-cell tolerance most likely mediated by clonal deletion, (ii) T-cell clonal ignorance, and (iii) nondeletional T-cell tolerance mediated by clonal anergy that is dependent on the structure, location, and concentration of the tolerogen. Secreted HBeAg was demonstrated to be significantly more efficient than intracellular HBcAg in eliciting T-cell tolerance.^74^ These findings highlight the critical role of the immune suppression function of HBeAg during breakthrough infection and the subsequent chronic progression of HBV infection.

The seropositivity of HBeAg is evident in approximately 90% of HBV carriers under six years of age, but can fall to 48% in carriers between 12 and 15 years of age. HBeAg-positive HBV carriers with overt liver dysfunctions in childhood are prone to lose HBeAg or to seroconvert to anti-HBe, followed by a marked histological regression.^75^ In Taiwan, HBV mutants with mutations in the precore stop codon coexisting with the wild-type strain were found in 10% of children initially and in 25% of children prior to HBeAg seroconversion. After HBeAg seroconversion, wild-type HBV was still present in 75% and the mutant HBV in 39% of the children. Children with earlier emergence of this mutant tended to have higher peak aminotransferase levels. Thus, this mutant is selected by host immune pressures.^76^ Interactions of host immunity with HBV replication in hepatocytes should be drivers of HBeAg seroconversion and persistent infection because the infection is established in early childhood.

## EFFECT OF GENDER ON THE CHRONIC PROGRESSION OF HBV INFECTION ACQUIRED IN EARLY CHILDHOOD

Chronic HBV infection occurs more frequently in male than in female HBV-infected children, although this gender tendency may not be as prominent as that observed in HBV-induced HCC patients. In most human populations there is a higher prevalence of male chronic HBV carriers compared to females, and the liver diseases caused by chronic HBV infection are more prevalent among male carriers. Interestingly, couples in which either parent is an HBsAg carrier have a higher proportion of male offspring compared with couples in which the parents are HBsAg-negative.^77^ The difference observed in chronic carriage between males and females is apparently due to a difference in the susceptibility of the two sexes to the development of the chronic carrier state: HBV infections before two years of age lead to chronic carriages of 77% in males and 50% in females.^78^ The prevalence of HBsAg is higher in males than in females from 10 to 29 years of age, and the prevalence of anti-HBs is higher in males up to age 60.^79^ Thus, we propose that sex hormones, their receptors, and the related signaling pathways are involved in immune imbalance that can lead to chronic HBV infection, hepatic inflammation, and the occurrence of subsequent end-stage liver diseases, such as HCC.

## EFFECT OF THE HOST GENETIC PREDISPOSITION ON THE CHRONIC PROGRESSION OF HBV INFECTION ACQUIRED IN EARLY CHILDHOOD

HBV genotypes and subgenotypes have distinct geographical distributions worldwide and have been shown to differ with regard to clinical diseases and responses to interferon treatment.^72,80^ Different human races have different HBV genotypes/subgenotypes. The HBV genotypes and subgenotypes prevalent in human races may reflect the long-term evolutionary history of family members of *hepadnaviridae*, including the “mutation-selection-adaptation” of HBV genotypes in different human races with specific immunogenetic backgrounds. Furthermore, HBV genotyping can be used to trace human immigration prior to the history of civilization.^72,81^ The question remains as to why different races have different prevalences of chronic HBV infection. For instance, the prevalence of HBsAg was as high as 10% in the Chinese population prior to the initiation of the HBV vaccination program but was less than 1% in the Caucasian population.^1,6^ Differences in the prevalence of chronic HBV infection in different populations depends on the epidemic background, the lifestyles of the affected residents, and the immunogenetic background of given races, of which single nucleotide polymorphisms (SNPs) of key immune/proinflammatory molecules play key roles in the chronic progression of HBV infection. Different human races may possess different patterns of SNPs that are responsible for different genetic susceptibilities to the chronic progression of HBV infection. As shown in Table 5, SNPs affecting the function of human leukocyte antigen (HLA) class II genes, including *HLA-DP* and *HLA-DQ*, as well as the inhibitory component of the nuclear factor-kappaB (NF-κB) complex IκBα gene *NFKBIA* are significantly associated with the chronic progression of HBV infection. The genetic loci whose rare alleles are significantly associated with decreased risks of chronic progression of HBV infection (or whose dominant alleles are significantly association with increased risks of chronic progression of HBV infection) include rs3138053 (*NFKBIA*), rs2856718, rs7453920, and rs9275319 (*HLA-DQ*), and rs9277378, rs2395309, rs2301220, and rs9277341 (*HLA-DP*); these loci counterchanged between the Han Chinese and European populations (Table 5) based on published studies.^82,83,84,85,86,87,88,89,90,91,92^ These data indicate that the Han Chinese are inherently more apt to progress into chronic infection once exposed to HBV than Europeans, whereas European tend to recover from HBV infection spontaneously.

Based on the International HapMap Project (http://www.hapmap.org/), the allelic distribution of the above SNPs in Japanese and Korean populations are similar to those in Han Chinese populations. However, the prevalence of HBsAg is approximately 2.5% in Japanese of the new generation and 4.0% in South Koreans after adjustment for age, sex, and geographic area.^93,94^ The prevalence of chronic HBV infection may also related to the epidemic background, life styles, and public health infrastructures that provide for safe medical practices. Unsafe infections, close contact with HBeAg-positive mothers and other family members with HBV infection, and close contact with children carrying HBV might be the major causes of the chronic progression of HBV infection acquired in early childhood or in adulthood.^62,63,64,65,66,67^ Currently, the young generation of Chinese born after 1992 in mainland China has a very low prevalence of HBsAg.^1^ This achievement is not completely attributable to the widespread application of HBV vaccination because the prevalence of anti-HBc antibody is also very lower in this population. The low prevalence of anti-HBc antibodies indicates that the rate of HBV infection is also lower in this population. Thus, public health prophylaxis plays an important role in decreasing the prevalence of chronic HBV infection.

## SUMMARY

A total of 42.1% of infants without HBV passive–active immunoprophylaxis born to HBsAg-carrier mothers globally and 2.9% of infants who received the immunoprophylaxis acquired HBV infection perinatally. Transplacental transmission, perinatal infection without HBV vaccination, and perinatal infection with the immunoprophylaxis occurred in 21.9% (14.3%–39.8%), 84.2% (18.8%–100%), and 8.7% (0.0–21.0%) of infants born to HBeAg-positive mothers, respectively; by contrast, the corresponding percentages were 11.7% (0.0–23.8%), 6.7% (0.0–15.4%), and 0.4% (0.0–2.5%) in infants born to HBeAg-negative-carrier mothers, respectively. The chronicity rates of perinatal HBV infection were 28.2% (17.4%–33.9%) in infants born to HBeAg-negative-carrier mothers and 64.5% (53.5%–100%) in infants born to HBeAg-positive-carrier mothers. Breakthrough infection mostly occurred in infants born to HBeAg-positive mothers, who usually had a viral load >10^6^ copies/mL. HBV MTCT was more frequent in East Asia compared to other areas. In addition to differences in HBV genotype endemicity, the interchange of allelic dominance of genetic polymorphisms in HLA class II (rs9277378, rs2395309, rs2301220, rs9277341, rs2856718, rs7453920, and rs9275319) and NF-κB (rs3138053) between Chinese and European populations may help to explain why chronic HBV infection occurs more frequently in Chinese than in European populations. The risk of progressing into chronic infection was inversely related to the age at which the children acquired the infection horizontally. In addition to MTCT, unsafe infections, close person-to-person contact with carriers (especially HBeAg-positive mothers) and incomplete vaccination may facilitate HBV transmission in endemic areas. To further diminish HBV chronic infection, it is necessary to enforce antiviral treatment after the 28th week of gestation for HBeAg-positive mothers and to improve health habits of carrier mothers, household sanitary conditions, and vaccination coverage, including unaffected adults.

## Figures and Tables

**Table 1:** Effect of the vaccination programmed on MTCT of HBV infection in infants born to HBsAg-carrier mothers seropositive or negative for HBeAg

**Status**	**Years of sample collection**	**Transplacental transmission, *n* (%)**	**HBIG**	**HBsAg-positive infants of six months to one year of age, *n* (%)**	**Nationality**	**Refs.**
**Without vaccination program**						
Maternal HBeAg-negative						
	1970–1974	–	No	2/13 (15.4)	Japanese	^48^
	1998–1990	–	No	3/49 (6.1)	Korean-American	^29^
	1983	–	No	0/13 (0.0)	American	^18^
Subtotal				5/75 (6.7, 0.0–15.4)		
Maternal HBeAg-positive						
	1970–1974	–	No	10/10 (100)	Japanese	^48^
	1998–1990	–	No	17/17 (100)	Korean-American	^29^
	–	–	No	140/159 (88.0)	Chinese	^11^
	1977–1980	–	–	3/16 (18.8)	West African	^17^
Subtotal				170/202 (84.2, 18.8–100)		
Unknown						
	1972–1973	–	No	63/158 (39.9)	Chinese	^24^
	1981–1983		–	7/17 (41.2)	Philippine	^33^
	–	19/40 (47.5)	–	5/17 (29.4)	Chinese	^49^
	2011–2012	36/50 (72.0)	No	12/50 (24.0)	Nigerian	^20^
	–	6/19 (31.6)	No	1/15 (6.7)	Turkish	^50^
	–	23/141 (16.3)	No	0/37 (0.0)	European	^51^
	–	10/54 (18.5)		0/54 (0.0)	Saudi	^52^
Subtotal		94/304 (30.9, 16.3–72.0)		88/348 (25.3, 0.0–41.2)		
**Total**		94/304 (30.9)		263/625 (42.1)		
**With vaccination program**						
Maternal HBeAg-negative						
	2009–2013	79/376 (21.0)	Yes	0/299 (0.0)	Chinese	^12^
	2007–2010	0/382 (0.0)	Yes	0/385 (0.0)	Chinese	^39^
	2007–2009	–	Yes	3/723 (0.4)	Chinese	^38^
	2007–2009	–	No	1/1050 (0.1)	Chinese	^38^
	2002–2005	–	Yes	4/163 (2.5)	Chinese	^40^
	1997–2002	–	Yes	0/63 (0.0)	Korean	^42^
	2009–2013	–	Yes	2/113 (1.8)	Singaporean	^53^
	2004–2009	–	Yes	3/201 (1.5)	Iranian	^43^
	1990–1995	0/20 (0.0)	Yes	0/20 (0.0)	American	^54^
	–	24/101 (23.8)	–	0/101 (0.0)	Greek	^35^
Subtotal		103/879 (11.7, 0.0–23.8)		13/3118 (0.4, 0.0–2.5)		
Maternal HBeAg-positive						
	2009–2013	64/161 (39.8)	Yes	11/144 (7.6)	Chinese	^12^
	2008–2012	24/78 (30.8)	Yes	–	Chinese	^55^
	2007–2010	69/482 (14.3)	Yes	27/484 (5.6)	Chinese	^39^
	2007–2009	–	Yes	54/583 (9.3)	Chinese	^38^
	2002–2005		Yes	6/41 (14.6)	Chinese	^40^
	1997–2002	–	Yes	17/81 (21.0)	Korean	^42^
	2009–2013	–	Yes	2/41 (4.9)	Singaporean	^53^
	2004–2009	–	Yes	6/34 (17.6)	Iranian	^43^
	–	3/8 (37.5)	–	0/8 (0.0)	Greek	^35^
Subtotal		160/729 (21.9, 14.3–39.8)		123/1416 (8.7, 0.0–21.0)		
Unknown						
	2000–2001	23/95 (24.2)	Yes	7/95 (7.4)	Chinese	^41^
	2006–2010	145/1360 (10.7)	Yes	21/1360 (1.5)	Chinese	^56^
	1984–1993	–	Yes	16/665 (2.4)	Chinese	^57^
	–	–	Yes	9/159 (5.7)	Chinese	^11^
	2004–2009	55/149 (36.9)	–	9/213 (4.2)	Indian	^58^
	–	2/73 (2.7)	Yes	4/58 (6.9)	French	^59^
Subtotal		225/1677 (13.4, 2.7–36.9)		66/2550 (2.6, 1.5–7.4)		
**Total**		488/3285 (14.8)		202/7084 (2.9)		

**Table 2:** Chronic progression of HBV infection acquired perinatally

**Maternal status**	**Years**	**Vaccination**	**Cases of children infect HBV, *n***	**Age started**	**Follow-up time (year)**	**Chronic, *n* (%)**	**Nationality**	**Refs.**
HBeAg-negative								
	2007–2009	Yes	23	–	0.5–10	4 (17.4)	Chinese	^38^
	1981–1982	Yes	68	6 months	1.5	15 (22.1)	American	^60^
	1990–2011	Yes	115	–	1–30	39 (33.9)	Iranian	^61^
Subtotal			206			58 (28.2, 17.4–33.9)		
HBeAg-positive								
	1984–1993	Yes	12	6 months	5–12	12 (100)	Chinese	^57^
	2007–2009	Yes	86	–	0.5–10	46 (53.5)	Chinese	^38^
	1981–1982	Yes	37	6 months	1.5	25 (67.6)	American	^60^
	1990–2011	Yes	24	–	1–30	18 (75.0)	Iranian	^61^
	1980–1982	No	27	Birth	1	19 (70.4)	Singaporean	^26^
Subtotal			186			120 (64.5, 53.5–100)		
Unknown								
	1972–1973	No	38	6 months	0–1.5	35 (92.1)	Chinese	^24^
	2006–2010	Yes	145	Birth	0.5–1	21 (14.5)	Chinese	^56^
	2004–2009	Yes	55	Birth	0.5–6	9 (16.4)	Indian	^58^
	2002–2005	Yes	10	Birth	2–3	2 (20.0)	Chinese	^40^
	1981–1983	No	17	Birth	0–1	7 (41.2)	Philippine	^33^
Subtotal			265			74 (27.9, 14.5–92.1)		
Total			657			252 (38.4)		

**Table 3:** Chronic progression of HBV infection acquired after birth

**Status**	**Years**	**HBV infection, *n***	**Follow-up time (year)**	**Chronic progression, *n* (%)**	**Nationality**	**Refs.**
Age group (year)						
0–4						
	1971–1976	21	6	6 (28.6)	American	^64^
	1977–1980	9	Up to 3	7 (77.8)	African	^17^
	1971–1975	80	0.5	17 (21.3)	Polish	^68^
5–9						
	1971–1976	61	6	10 (16.4)	American	^64^
10–18						
	1971–1976	58	6	4 (6.9)	American	^64^
Total		229		44 (19.2)		

**Table 4:** Effect of the HBV vaccination programmed on the ratios of vertical transmission and horizontal transmission in subjects with chronic infection

	**Years**	**Cases, *n***	**Vertical**	**Horizontal**	**Not sure**	**Nationality**	**Refs.**
Without vaccination program							
	1950	522,500	197,574 (37.8)	324,926 (62.2)	–	Japanese	^69^
	1966–1984	158	63 (39.9)	95 (60.1)	–	Chinese	^47^
	1979–1980	669	141 (21.0)	528 (79.0)	–	Chinese	^19^
	1985	486,038	185,871 (38.2)	300,168 (61.8)	–	Japanese	^69^
Subtotal		1,009,365	383,649 (38.0, 21.0–39.9)	625,472 (62.0, 60.1–19.0)			
With vaccination program							
	1999	48	39 (81.3)	9 (18.7)	–	Chinese	^47^
	1983–2008	252	151 (59.9)	37 (14.7)	64 (25.4)	Asian	^70^
	2000–2009	570	210 (36.8)	70 (12.3)	270 (47.4)	Turkish	^71^
Subtotal		870	400 (46.0, 36.8–81.3)	116 (13.3, 12.3–18.7)			

**Table 5:** Association of single nucleotide polymorphisms (SNPs) affecting the functioning of immune/proinflammatory molecules with HBV persistence

							**Dominant allele^a^, *n* (%)**		
**Gene**	**Locus**	**SNPs**	**Comparable groups**	**OR (95% CI)**	***P* value**	**Nationality**	**Han Chinese**	**European**	**Refs.**
NFKBIA	rs3138053	G/AA	HBV vs. clearance	0.54 (0.34, 0.86)	0.01	Chinese	A (82.6)	G (50.0)	^82^
HLA-DQ	rs2856718	GG/AA	HBV vs. clearance	0.59 (0.47, 0.73)	<0.001	Chinese	A (53.3)	G (73.3)	^83^
		G/A	HBV vs. healthy controls	0.64 (0.59, 0.69)	<0.001	Japanese			^84^
HLA-DQ	rs7453920	AG/GG	HBV vs. clearance	0.62 (0.50, 0.76)	<0.001	Chinese	G (88.4)	G (52.2)	^83^
		A/G	HBV vs. healthy controls	0.55 (0.36, 0.85)	<0.001	Japanese	G (81.4) for Japanese		^84^
HLA-DQ	rs9275319	AG/AA	HBV vs. clearance	0.63 (0.47, 0.85)	0.003	Chinese	A (81.4)	G (50.0)	^85^
HLA-DP	rs3077	A/GG	HBV vs. clearance	0.72 (0.56, 0.92)	0.008	Chinese	G (62.5)	A (88.3)	^86^
		A/G	HBV vs. healthy controls	0.53 (0.49, 0.58)	<0.001	Japanese	G (58.7) for Japanese		^84,87^
		AA/GG	HBV vs. clearance	0.51 (0.41, 0.62)	<0.001	Thai, Chinese			^83,88,89^
		G/A	HBV vs. healthy controls	5.1 (1.9–13.7)	<0.001	Caucasian			^90^
HLA-DP	rs9277535	A/GG	HBV vs. clearance	0.73 (0.57, 0.94)	0.0162	Chinese	G (53.5)	A (72.5)	^86^
		AA/GG	HBV vs. clearance	0.37 (0.31, 0.44)	<0.001	Chinese			^83,89^
		A/G	HBV vs. healthy controls	0.56 (0.52, 0.61)	<0.001	Japanese	G (58.0) for Japanese		^84,87^
		G/A	HBV vs. healthy controls	1.2 (0.5, 2.6)	1	Caucasian			^90^
HLA-DP	rs9277378	AA/GG	HBV vs. clearance	0.41 (0.20, 0.87)	0.018	Thai	G (53.4)	A (69.2)	^88^
HLA-DP	rs3135021	AA/GG	HBV vs. clearance	0.55 (0.36, 0.85)	0.007	Chinese	G (75.6)	G (75.0)	^89^
HLA-DP	rs2281388	T/CC	HBV vs. clearance	1.35 (1.05, 1.75)	0.021	Chinese	C (69.8)	C (97.5)	^86^
		TT/CC	HBV vs. clearance	3.09 (2.01, 4.74)	<0.001	Chinese			^89^
HLA-DP	rs2395309	AA/GG	HBV vs. clearance	0.42 (0.29, 0.62)	<0.001	Chinese	G (61.9)	A (83.2)	^89^
HLA-DP	rs2301220	GG/AA	HBV vs. clearance	0.40 (0.27, 0.59)	<0.001	Chinese	A (61.4)	G (88.3)	^89^
HLA-DP	rs9277341	TT/CC	HBV vs. clearance	0.30 (0.16, 0.60)	<0.001	Chinese	C (75.6)	T (73.2)	^89^
HLA-DP	rs10484569	GG/AA	HBV vs. clearance	2.80 (1.87, 4.20)	<0.001	Chinese	G (59.8)	G (94.2)	^89^
HLA-DP	rs3128917	GG/TT	HBV vs. clearance	3.53 (2.43, 5.12)	<0.001	Chinese	G (55.2)	T (69.2)	^89^
HLA-DP	rs3117222	TT/CC	HBV vs. clearance	3.68 (2.52, 5.36)	<0.001	Chinese	G (58.3)	G (74.8)	^89^
HLA-DP	rs9380343	TT/CC	HBV vs. clearance	3.12 (2.06, 4.73)	<0.001	Chinese	C (68.6)	C (92.5)	^89^
IL28B	rs12979860	T/C	Healthy controls vs. HBV	–	0.03	Korean	C (93.3)	–	^91^
IL28B	rs8099917	G/T	Healthy controls vs. HBV	–	0.009	Korean	T (90.7)	T (85.0)	^91^
IL10	rs1518110	TT/GG	HBV vs. clearance	0.11 (0.01, 0.93)	0.004	African American	–	G (73.9)	^92^
IL20	rs1518108	CC/TT	HBV vs. clearance	5.45 (1.31, 22.8)	0.02	African American	C (86.3)	C (69.0)	^92^
IL20	rs1400986	CC/TT	HBV vs. clearance	0.23 (0.07, 0.80)	0.02	European American	C (88.4)	C (86.3)	^92^
IL20	rs3024517	AA/GG	HBV vs. clearance	0.16 (0.04, 0.70)	0.01	European American	–	A (91.3)	^92^

a Dominant allele of SNPs in the Chinese Han population and European population were identified according to the International HapMap Project (http://www.hapmap.org/).
